# Tumor Control Probability Modeling for Radiation Therapy of Keratinocyte Carcinoma

**DOI:** 10.3389/fonc.2021.621641

**Published:** 2021-05-17

**Authors:** Phillip Prior, Musaddiq J. Awan, J Frank Wilson, X. Allen Li

**Affiliations:** Department of Radiation Oncology, Medical College of Wisconsin, Milwaukee, WI, United States

**Keywords:** tumor control probability (TCP), basal cell/carcinoma, squamous cell carcinama, biological effective dose (BED), dose response modeling

## Abstract

**Summary:**

Skin cancer patients may be treated definitively using radiation therapy (RT) with electrons, kilovoltage, or megavoltage photons depending on tumor stage and invasiveness. This study modeled tumor control probability (TCP) based on the pooled clinical outcome data of RT for primary basal and cutaneous squamous cell carcinomas (BCC and cSCC, respectively). Four TCP models were developed and found to be potentially useful in developing optimal treatment schemes based on recommended ASTRO 2020 Skin Consensus Guidelines for primary, keratinocyte carcinomas (*i.e.* BCC and cSCC).

**Background:**

Radiotherapy (RT) with electrons or photon beams is an excellent primary treatment option for keratinocyte carcinoma (KC), particularly for non-surgical candidates. Our objective is to model tumor control probability (TCP) based on the pooled clinical data of primary basal and cutaneous squamous cell carcinomas (BCC and cSCC, respectively) in order to optimize treatment schemes.

**Methods:**

Published reports citing crude estimates of tumor control for primary KCs of the head by tumor size (diameter: ≤2 cm and >2 cm) were considered in our study. A TCP model based on a sigmoidal function of biological effective dose (*BED*) was proposed. Three-parameter TCP models were generated for BCCs ≤2 cm, BCCs >2cm, cSCCs ≤2 cm, and cSCCs >2 cm. Equivalent fractionation schemes were estimated based on the TCP model and appropriate parameters.

**Results:**

TCP model parameters for both BCC and cSCC for tumor sizes ≤2 cm and >2cm were obtained. For BCC, the model parameters were found to be *TD_50_* = 56.62 ± 6.18 × 10^-3^ Gy, *k* = 0.14 ± 2.31 × 10^−2^ Gy^−1^ and *L* = 0.97 ± 4.99 × 10^−3^ and *TD_50_* = 55.78 ± 0.19 Gy, *k* = 1.53 ± 0.20 Gy^−1^ and *L* = 0.94 ± 3.72 × 10^−3^ for tumor sizes of ≤2 cm and >2 cm, respectively. For SCC the model parameters were found to be *TD_50_* = 56.81 ± 19.40 × 10^4^ Gy, *k* = 0.13 ± 7.92 × 10^4^ Gy^−1^ and *L* = 0.96 ± 1.31 × 10^-2^ and *TD_50_* = 58.44 ± 0.30 Gy, *k* = 2.30 ± 0.43 Gy^−1^ and *L* = 0.91± 1.22 × 10^−2^ for tumors ≤2cm and >2 cm, respectively. The TCP model with the derived parameters predicts that radiation regimens with higher doses, such as increasing the number of fractions and/or dose per fraction, lead to higher TCP, especially for KCs >2 cm in size.

**Conclusion:**

Four TCP models for primary KCs were developed based on pooled clinical data that may be used to further test the recommended kV and MV x-ray and electron RT regimens from the 2020 ASTRO guidelines. Increasing both number of fractions and dose per fraction may have clinically significant effects on tumor control for tumors >2 cm in size for both BCC and cSCC.

## Introduction

Skin cancer is the most common form of cancer in the United States with an annual incidence of five million cases (1). Keratinocyte carcinoma (KC) consists of basal and cutaneous squamous cell carcinomas (BCCs and cSCCs, respectively) are the most common forms of skin cancers [more commonly known as non-melanoma skin cancers (NMSC)] (2) and account for over 95% of skin cancer diagnoses (3). Definitive radiotherapy (RT) using kilovoltage (kV) x-rays, megavoltage electrons (MeV), and megavoltage (MV) x-rays, especially for deeply invasive KC, is an excellent treatment option for skin cancer, particularly as an alternative to surgery in non-operative candidates, when there are cosmetic concerns with surgery or when patients refuse surgery. Reported tumor control rates using definitive RT for BCCs and cSCCs are upwards of 90−95% and are comparable to surgical resection (4–6). However, the benefit of RT may depend on tumor size: control rates for smaller tumors (≤2 cm) greatly exceed 90% while control rates for large (>2 cm) may be as low as 60−70% (4–6). A variety of dose and fractionation schemes for definitive RT of BCCs and cSCCs are reported in the literature. The RT techniques used in these studies mostly pre-date the era of image guided radiation and thus employed large treatment margins. The ASTRO 2020 Skin Consensus Guidelines was published in early 2020 and recommended various treatment scenarios for NMSC that include RT delivery technique and fractionation considerations for primary RT and post-operative scenarios, *etc.* (3) The purpose of this work is to add to this recent report by modeling tumor control probability (TCP) based on available clinical outcome data of definitive RT kV photons, MV photons and MeV electrons for BCC and cSCC in order to optimize treatment schedules based on tumor size using modern conformal RT techniques.

## Materials and Methods

**Figure 1:**
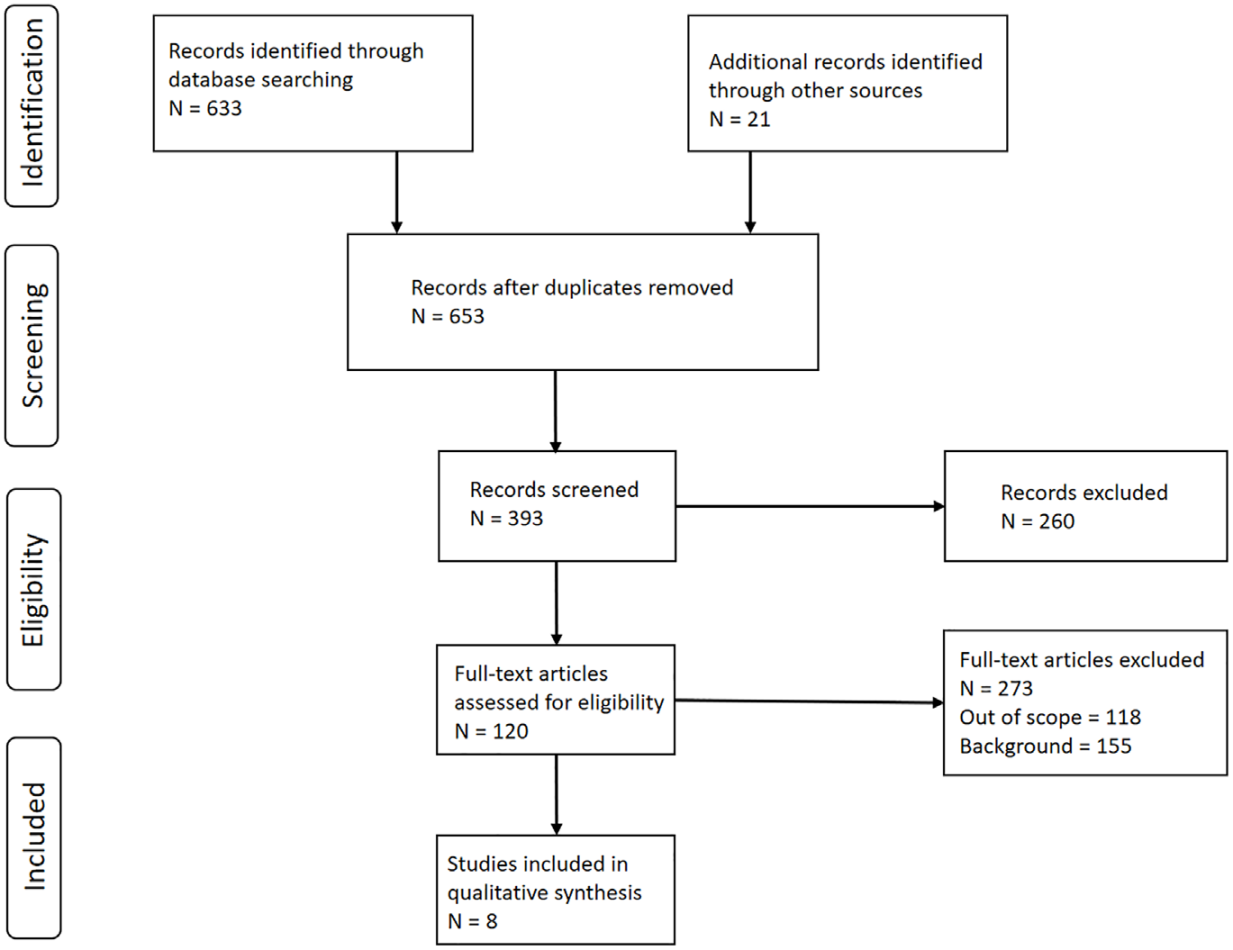
Preferred Reporting Items for Systematic Reviews and Meta-Analysis (PRISMA) Diagram. Schematic depicting the literature review process for the development of basal and cutaneous squamous cell carcinoma (BCC and cSCC, respectively) tumor control probability (TCP) models. Created based on Moher D, A Liberati, J Tetzlaff and DC Altman, 2009 [ref (7)].

A literature review was performed using the Preferred Reporting Items for Systematic Reviews and Meta-Analyses (PRISMA) procedure described by Moher et al. (7) (**Figure 1**) using the search criteria and the references within the recent ASTRO guidelines (3). A second search was subsequently performed using a PubMed search with search string “radiotherapy AND basal cell carcinoma AND squamous cell carcinoma,” and a date range from 1/1980 to 6/2020. Next, an additional search of articles referencing those articles found in the recent ASTRO guidelines and the PubMed search was subsequently performed. Reports citing crude estimates of primary tumor site recurrence rate (*RR, i.e.* number recurred in the field divided by the total number of BCC or cSCC patients) for primary BCCs and cSCCs of the head, head and neck region, or extremities by tumor size (*i.e.* tumor diameter) were considered for use in developing our TCP model. The modality considered in our analysis consisted of using kV photons available on superficial and orthovoltage machines, as well as MV photons and electrons typically available on linear accelerators. The TCP was subsequently calculated as *TCP^literature^* = *1 – RR*. Our model was based on the following phenomenological sigmoidal equation (8):

(1) $$TCP\left(TD_{50},k,L\right)=\frac{L}{1+e^{-\left[D\left(1+d/\frac{\alpha }{\beta }\right)-TD_{50}\right]/k}}$$

where *D* is the total prescribed dose, *d* is the dose per fraction (Gy/fx), *α* and *β* are linear quadratic (LQ) model radiobiological parameters (we use *α*/*β* = 10 Gy, in line with the ASTRO guidelines), *TD_50_* is the dose required to achieve 50% tumor control, *k* is a fitting constant related to the slope of the dose response curve at *TD_50_*, and *L* is a logistic function maximum value or level. The values for *D* and *d* used were the mean values reported in the manuscript for the patient cohort of each tumor type and size (*e.g.* BCC ≤2 cm in diameter). Our analysis considered *TD_50_*, *k* and *L* as fitting parameters and were estimated using the chi-squared fitting method by minimization of the following equation:

(2) $$\chi^{2}=\sum_{i=1}^{M}\frac{{\left(TCP_{i}^{model}\left(\;TD_{50},\;k,L\right)-TCP_{i}^{literature}\right)}^{2}}{\sigma_{i}^{2}},$$

where *M* = number of data points, $TCP_{i}^{model}$ and $TCP_{i}^{literature}$ are the *TCP* of the model and report, respectively, for the *i* ^th^ data point, while $\sigma_{1}^{2}$ is the statistical error in the *i* ^th^ data point. The statistical error was calculated using the following (9):

(3) $$\sigma_{RR,i}=RR_{i}^{literature}\sqrt{\frac{1-RR_{i}^{literature}}{N_{i}}},$$

(4) $$\sigma_{i}^{2}={\left(\frac{dTCP^{literature}}{dRR}\right)}^{2}\cdot \sigma_{RR,i}^{2},$$

where *N_i_* is the number of responders that did not recur for the *i^th^* data point (or alternatively the number of responders controlled at the time the data point was obtained), *σ_RR,i_*. is the uncertainty of the response rate of the *i^th^* data point, RR_literature_ is the response rate of the *i^th^* data point, *dTCP^literature^/dRR* is the derivative of the tumor control probability with respect to the recurrence rate (i.e. this quantity evaluates to −1). Optimization was performed and standard errors in parameter estimates were obtained using Matlab (MathWorks, Natick, MA). Additional analysis was performed by calculating the TCP for each of the four models using suggested dose-fractionation schemes from the recently published ASTRO Clinical Practice Guidelines using various treatment modalities including MeV electrons, kV photons and MV photons.

## Results

Literature search yielded a total of eight reports useful for the development of a TCP model for BCC and cSCC for which tumor control could be specified based on tumor size ≤2 cm and >2 cm (**Table 1**) (4, 5, 10–15). Seven of these reports used orthovoltage and megavoltage photons/electrons for treatment (3, 4, 6–8), while van Hezewijk et al. exclusively used electrons (13). All of these reports provided response data for tumor sizes ≤2 cm and seven reports provided data for tumors >2 cm. The majority of the 120 reports were not included in the final analysis because they didn’t specify the radiation treatment schedule for their patient cohort by tumor size (*i.e.* tumor stage). If it was specified, that schedule was stated for the entire cohort and not further broken down by tumor size. The last major reason for rejection was that the report only specified treatment outcomes and radiation schedule for the entire patient population nor was it possible to discern the characteristics of the patient outcome and radiation schedule by tumor size.

**Table 1:** Description of studies used in the development of a tumor control probability (TCP) model for basal and cutaneous squamous cell carcinomas (BCC and cSCC, respectively) by tumor size.

Report	Path. Type	*T* (days)	*D* (Gy)	*d* (Gy)	BED_10_ (Gy)	FU(yrs)	Total ≤2 cm	Number Recurred & ≤2 cm	Total >2cm	Number Recurred & >2 cm
**Cognetta et al. (** 4 **)**	BCC	10.0	35.0	7.0	59.50	2.6^#^	712	22		
	cSCC	10.0	35.0	7.0	59.50	2.6^#^	994	23		
**Hernandez-Machin et al. (** 10 **)**	BCC	28.4	40.9	7.3	70.76	4.9^#^	571	29	43	3
	cSCC	28.5	42.4	6.7	70.81	4.8^#^	101	6	10	1
**Locke et al. (** 5 **)**	BCC	33.0	48.5	2.6	61.11	5.8	197	7	56	5
	cSCC	39.1	52.9	2.4	65.60	5.8	41	2	28	4
**Petrovich et al. (** 11 **)**	BCC	18.4	43.2	3.2	57.02	7.5^#^	432	24	32	10
	cSCC	18.4	43.2	3.2	57.02	7.5^#^	170	19	12	4
**Schulte et al. (** 12 **)**	BCC	26.1	61.0	4.7	94.65	6.8	615	15	388	21
	cSCC	25.2	63.1	5.0	94.65	6.8	92	2	152	15
**van Hezewijk et al. (** 13 **)**	BCC	20.7	47.8	3.9	66.44	3.6	240	6	92	4
	cSCC	27.0	50.8	3.5	68.58	3.6	46	1	56	4
**Duinkerken et al. (** 14 **)**	BCC	15.9	54.1	3.4	72.49	1.9	183	3	33	2
**Terra et al. (** 15 **)**	cSCC	22.34	57.9	2.6	72.95	1.9	52	4	52	4
**Average**	BCC	21.8	47.2	4.6	69.87	4.7	2844*	106*	644*	45*
	SCC	24.4	49.3	4.3	68.14	4.7	1439*	57*	310*	32*

Path. Type, pathology type of tumor; T, mean treatment time reported in the manuscript for the patient cohort’s tumor type and size; D, mean total dose reported in the manuscript for the patient cohort’s tumor type and size; d, mean dose per fraction reported in the manuscript for the patient cohort’s tumor type and size; FU (yrs), median follow-up in years reported in the manuscript except where denoted by the # symbol; Total ≤2 cm, total number of patients with tumor sizes ≤2 cm; number Recurred & ≤2 cm, the number of patients that did recur with tumor sizes ≤2 cm; Total >2 cm, total number of patients with tumor sizes >2 cm; Number Recurred & >2 cm, the number of patients that did recur with tumor sizes >2 cm; *denotes total number of patients or responders; and ^#^denotes the average follow-up of patients reported in the manuscript.

The numbers of recurrences were found to be 125 of 3,594 for BCC tumors ≤2 cm and 45 of 644 for BCC tumors >2 cm recurred. For the treatment of BCCs, the reported mean total radiation dose was 47.2 Gy (range: 35–61 Gy), the mean dose per fraction was 4.6 Gy/fx (range: 2.6–7.3 Gy/fx), [biological effective dose (*BED*) using LQ model with *α*/*β* = 10 Gy (*BED_10_*)] *BED_10_* = 69.9 Gy, (range: 57.0–94.7 Gy)], and the mean total treatment time was 21.8 days (range: 10.0–39.1 days). The average follow-up time was 4.7 years (range: 1.9–7.5 years).

The number of recurrences was found to be 57 of 1,806 for cSCC tumors ≤2 cm, and 36 of 284 for cSCC tumors >2 cm recurred. For the treatment of cSCCs, the reported mean total dose was 49.3 Gy (range: 35–63.1 Gy), the mean dose per fraction was 4.3 Gy/fx (range: 2.4–7.0 Gy/fx), [*BED_10_* = 68.1 Gy, (range: 59.5–94.7 Gy)], and mean total treatment time was 23.5 days (range: 6.0–39.2 days). The average follow-up time was 4.7 years (range: 1.9–7.5 years).

**Figure 2:**
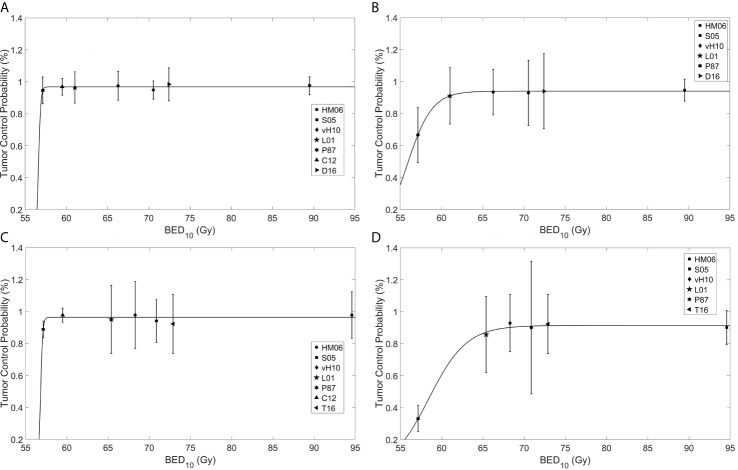
Fitting of a tumor control probability (TCP) model to reported clinical data for basal cell carcinoma (BCC) of size **(A)** ≤2 cm and **(B)** >2 cm, and cutaneous squamous cell carcinoma (cSCC) of size **(C)** ≤2 and **(D)** >2 cm. The abscissa is the biological effective dose calculated using the linear quadratic model with *α*/*β* = 10 Gy (BED_10_), while the ordinate is the TCP. HM06, Hernández-Machin et al., 2006 (10) (circle); S05, Schulte et al., 2005 (12) (square); VH10, van Hezewijk et al., 2010 (13) (diamond); L01, Locke et al., 2001 (5) (pentagram); P87, Petrovich et al., 1987 (11) (hexagram); Co12, Cognetta et al., 2012 (4) (upward pointing triangle); D16, Duinkerken et al., 2016 (14) (right pointing triangle); T16, Terra et al., 2016 (15) (left pointing triangle).

Four sets of model parameters for the TCP model (Eq. 1) were generated for (BCC and cSCC stratified by tumor size using a 2 cm cutoff (BCC ≤2cm, BCC >2cm, cSCC ≤2 cm, and cSCC >2 cm) (**Table 2**) assuming an *α*/*β* = 10 Gy. For BCC, the model parameters were found to be *TD_50_* = 56.62 ± 6.18 × 10^−3^ Gy, *k* = 0.14 ± 2.31 × 10^−2^ Gy^−1^ and *L* = 0.97 ± 4.99 × 10^−3^ and *TD_50_* = 55.78 ± 0.19 Gy, *k* = 1.53 ± 0.20 Gy^−1^ and *L* = 0.94 ± 3.72 × 10^−3^ for tumor sizes of ≤2 cm and >2 cm, respectively. For SCC the model parameters were found to be *TD_50_* = 56.81 ± 19.4 × 10^4^ Gy, *k* = 0.13 ± 7.92 × 10^4^ Gy^−1^ and *L* = 0.96 ± 1.31 × 10^−2^ *and TD_50_* = 58.44 ± 0.30 Gy, *k* = 2.30 ± 0.43 Gy^−1^ and *L* = 0.91 ± 1.22 × 10^−2^ for tumors ≤2 cm and >2 cm, respectively. The TCP response curves for BCC and cSCC tumors are depicted in **Figure 2**.

**Table 2:** Tumor control probability (TCP) model parameters for basal and cutaneous squamous cell carcinomas (BCC and cSCC, respectively) tumors of size ≤2 and >2 cm.

Path. Type	*TD_50_ (Gy)*	*k (Gy^-1^)*	*L*
**BCC ≤2 cm**	56.62 ± 6.18 × 10^−3^ 95% CI (56.61–56.63)	0.14 ± 2.31 × 10^-2^ 95% CI (0.094–0.098)	0.97 ± 4.99 × 10^-3^ 95% CI (0.96-0.98)
**BCC >2 cm**	55.78 ± 0.19 95% CI (55.18–56.37)	1.53 ± 0.20 95% CI (0.91-2.16)	0.94 ± 3.72 × 10^-3^ 95% CI (0.93–0.95)
**SCC ≤2 cm**	56.81 ± 1.94 × 10^4^ 95% CI (-380896–381009.7)	0.13 ± 7.92 × 10^4^ 95% CI (−155210–155210.7)	0.96 ± 1.31 × 10^-2^ 95% CI (0.94–0.99)
**SCC >2 cm**	58.44 ± 0.30 95% CI (57.48–59.41)	2.30 ± 0.43 95% CI (0.93–3.66)	0.91 ± 1.22 × 10^-2^ 95% CI (0.88–0.95)

95% CI, 95% confident interval.

The TCP model was used to calculate the *BED_10_* of different fractionation schemes (**Table 3**, reported in Appendix KQ4 of Likhacheva et al.). For example, a TCP of 95% or more for smaller sized tumors (*i.e*. ≤2 cm) could be achieved using a fractionation scheme of 15 treatments of 3 Gy per fraction (Gy/fx), regardless of tumor type. In contrast, larger sized tumors show lower tumor control for the same *BED_10_* using the recommended fractionation schemes in Likhacheva et al. (*e.g.* 20 treatments of 2.35 Gy/fx). In contrast to smaller tumors, for tumors >2 cm, TCP varied by histological type; BCC tumors have higher TCP values compared to cSCC tumors of the same size and fractionation schedule (*e.g. TCP_BCC>2_* = 0.765 and *TCP_SCC>2_*= 0.417 for 20 treatments of 2.35 Gy/fx).

**Table 3:** Tumor Control Probability (TCP) for different radiation schedules by tumor type and size.

Fractionation Schedule (*N* × *d*)	*BED_10_* (Gy)	TCP_BCC ≤2_	TCP_BCC>2_	TCP_cSCC ≤2_	TCP_cSCC>2_
25 × 2.00	60.0	0.968	***0.884***	0.964	***0.606***
30 × 2.00	72.0	0.968	0.940	0.964	0.912
20 × 2.35	58.0	0.968	***0.765***	0.964	***0.417***
15 × 3.00	58.5	0.968	***0.804***	0.964	***0.463***
16 × 3.00	62.4	0.968	0.928	0.964	***0.776***
20 × 3.00	78.0	0.968	0.940	0.964	0.914
15 × 4.00	84.0	0.968	0.940	0.964	0.914
10 × 4.20	59.6	0.968	***0.870***	0.964	***0.573***
8 × 5.00	60.0	0.968	***0.884***	0.964	***0.606***
10 × 5.00	75.0	0.968	0.940	0.964	0.913

N, number of fractions; d, dose per fraction (Gy/fx); BED_10_, biological effective dose calculated using linear quadratic (LQ) model with α*/*β = 10 Gy; TCP_BCC_ ≤ _2_, TCP for basal cell carcinoma (BCC) tumors of size ≤2 cm; TCP_BCC>2_, TCP for BCC tumors of size >2 cm; TCP_SCC_ ≤ _2_, TCP for cutaneous squamous cell carcinomas (cSCC) tumors of size ≤2 cm; and TCP_SCC>2_, TCP for cSCC tumors of size >2 cm. Those TCP below 0.900 are depicted in the table in bold, italicized font.

## Discussion

The projected increase in KC (formerly known as NMSC) incidence necessitates the development of outcome-based models to help design optimal schemes for RT treatment planning and clinical trials to further investigate the dose response relationship with tumor stage (1, 2). The TCP model along with appropriate parameters is the first to our knowledge to be developed for BCCs and cSCCs stratified by tumor size. This model was developed using crude estimates of tumor control from a literature review of patient populations treated with primary definitive RT for BCCs and cSCCs. Therefore, the model is not applicable in designing treatment schedules for patients treated for recurrent disease or in the post-operative setting. Additionally, the model would not be applicable for treatment sites other than the head and neck region since recurrent BCC and cSCC risk factors were developed using reports in the anatomical region (3). Nor would the model be applicable in situations where the appropriate margins were applied in creating the target volume [*i.e.* planning target volume (PTV)] for treatment (3, 16). However, our model would be useful in designing future prospective and randomized trials of KC in order to characterize the role of definitive RT in the management of this disease following the guidelines set forth in Likhacheva et al. (3).

Our model utilizes a phenomenological-based function for TCP with a small number of parameters that’s consistent with the KC clinical data, which could be viewed as a weakness. However, this is in contrast to a Poisson-based model, which would have more parameters leading to an overfitting of the data and have less predictive power given the small number of reports published over a large time period (17). Additionally, our models used the LQ model-derived BED to compare different RT fractionation regimes rather than physical dose and assumed an *α*/*β* of 10 Gy, which is consistent with the dose-fractionation analysis in the recent ASTRO guidelines (3). This seemed reasonable considering that Trott et al., Dale & Thomas et al. reported 95% confidence intervals in their estimates of KC *α*/*β* that included or were within 0.5 Gy of 10 Gy (18–20). We did not consider time in our model since Fitzpatrick et al. suggest that a reduction in total *BED* over the course of 1–2 months is insignificant for BCC and cSCC tumors (6). The reported models in our report may be useful in designing more comprehensive clinical trials to better illuminate dose–response by tumor size and/or stage, as well as develop more predictive TCP models of KC.

There are sources of uncertainty and other limitations to our modeling approach. The basis of much of the literature review was based on the ASTRO 2020 skin cancer guidelines, which used a time period of May 1988 to June 2018 on non-metastatic BCC and cSCC patients treated to curative intent (3). Our use of a wider time frame with basic keywords, as well as reviewing papers citing or cited by the references included in the guidelines, was to catch any manuscripts that may not have been included in the ASTRO consensus guidelines, which are literature search found two not included (11, 15). The patient population included KC patients treated definitively with kV photons, MeV electrons, and MV photons and excluded patients treated with high dose rate (HDR) brachytherapy despite the latter having similar local control and cosmetic outcomes as the former (3). Our rationale is that our model would be used as the basis of a clinical trial investigating the dose response of KCs by tumor stage, and kV and MV photons and MeV electrons are suggested for treating all tumor stages and commonly available on linear accelerators (MV photons and MeV electrons) and orthovoltage superficial machine (kV photons) that are used in radiation oncology and dermatological clinics (3). However, brachytherapy isn’t suggested for high T stage (hence large sized) tumors nor is it as commonly used as linear accelerators (3). Though with skin cancer rates projected to rise in the future, Cognetta et al. showed the kV photons could be useful tool in the dermatology clinic’s management of skin cancer in the increasing elderly and frail patient population (4). The wide range of follow-up times (*i.e.* 1.9–7.5 years) is a source of uncertainty in our analysis. The patient cohort in studies with shorter follow-up times may not have had sufficient time to reach stable outcome, particularly those with higher stage tumors (21). The use of the prescribed dose in our analysis represents another source of uncertainty given the reported associations of the minimum target dose and tumor control (22). Increasing tumor control by way increasing *D* and *d* should be done without exceeding normal tissue tolerances. Disregarding of normal tissue doses in the quest for improved tumor control could cause unacceptable toxicity as has been found in Hodgkin’s Lymphoma (23).

## Conclusion

TCP models along with appropriate model parameters for primary BCC and cSCC tumors of size ≤2 cm and >2 cm were derived based on the best available clinical data, which may be used to develop alternative treatment schedules accounting for tumor size and utilizing modern RT technologies. The TCP calculations suggest that the recommended fractionation schedules in the ASTRO guidelines may be optimized to improve local control for tumor sizes larger than 2 cm. Based on the present work, larger sized BCC and cSCC tumors may be treated with higher fractionated doses. For example, for BCCs >2 cm, 16 (or more) treatments of 3 Gy/fx (or higher) in order to achieve a TCP of ~90% or higher. Similarly, cSCC tumors >2 cm, 20 or more treatments of 3 Gy in order to achieve a TCP of 90% or higher.

## Data Availability Statement

The raw data supporting the conclusions of this article will be made available by the authors, without undue reservation.

## Author Contributions

MA and JW provided clinical expertise on non-melanoma skin cancer. PP and XL developed clinically relevant TCP model in consultation with MA and JW. All authors contributed to the article and approved the submitted version.

## Conflict of Interest

The authors declare that the research was conducted in the absence of any commercial or financial relationships that could be construed as a potential conflict of interest.
